# Barriers to belonging: an ecological and appraisal framework for peer relationships, self-awareness, and confidence in deaf and hard of hearing

**DOI:** 10.3389/fpsyg.2025.1632263

**Published:** 2025-10-08

**Authors:** Abdulrahman Alsayed

**Affiliations:** Department of Special Education, King Faisal University, Al-Ahsa, Saudi Arabia

**Keywords:** social and emotional development, d/Deaf and hard of hearing, ecological systems theory, appraisal theory of emotion, peer relationships, self-awareness, confidence

## Abstract

This systematic review explores the social and emotional development (SED) of Deaf or Hard of Hearing (DHH) individuals, guided by Ecological Systems Theory and the Appraisal Theory of Emotion. These frameworks highlight how language access, identity, communication methods, and environmental factors influence key areas of SED, including emotional regulation, peer relationships, self-awareness, and confidence. Following PRISMA guidelines, a comprehensive search was conducted across seven databases, yielding 2,380 records. After removing duplicates and applying inclusion and exclusion criteria, 23 empirical studies published between 2010 and 2025 were included in the final review. The studies used quantitative (65.2%), qualitative (26.1%), and mixed-methods (8.7%) designs and represented a range of age groups, communication modalities, and geographic settings. Most focused on children and adolescents, with additional research involving adults and preschool-aged individuals. The findings emphasize the importance of inclusive educational settings, consistent language access (signed or spoken), and culturally affirming support in promoting positive SED outcomes for DHH individuals. This review underscores the need to integrate social–emotional goals into educational and clinical practice and encourages interdisciplinary efforts that recognize both the challenges and strengths shaping the development of DHH individuals.

## Introduction

Social and Emotional Development (SED) is fundamental, as well-developed social–emotional skills are key drivers of emotional well-being, meaningful relationships, and long-term success in both personal and professional life (Atkins et al., 2023; Calderon and Greenberg, 2011; Dowling, 2014; Ha, 2023; Hintermair et al., 2017; Umberson and Montez, 2010). SED have been the focus of research in different fields such as psychology, health science, and education. However, researchers in these various fields use different terms to describe SED for an individual. The most common terms include social–emotional development (Ashdown and Bernard, 2012), social–emotional competence (Bierman et al., 2014), and social–emotional skills (Humphrey et al., 2011). This paper will use the term SED to explain three important skills: developing a relationship with peers, self-awareness, and confidence. These three SED skills will be connected to the Ecological Systems Theory and the Appraisal Theory of Emotion. SED are crucial in early childhood development, and cognitive development cannot be isolated from social and emotional development (Thompson and Goodman, 2009). In school-age children, SED are the foundation for appropriate academic development (Boyd et al., 2005). SED are associated with the development of multiple skills, including cognitive skills and language development, which directly affect how individuals feel about themselves and their expression of emotions (Zero to Three, 2016). It is imperative to notice the importance of SED during early childhood years and even later as a child attends school.

The literature has shown the majority of Deaf and Hard of Hearing (DHH) students demonstrate the same intelligence as their hearing peers (Brown and Cornes, 2015; Maller and Braden, 2011). However, communication and language development for students who are DHH impact their SED (Antia et al., 2011). Therefore, research in the field suggests that DHH students are more likely to experience social–emotional difficulties than their hearing peers (Antia and Kreimeyer, 2015; Batten et al., 2013; Mekonnen et al., 2015; Punch and Hyde, 2011; Rieffe, 2012). As the research showed previously, emotional competencies are associated with different skills such as academic and language and communication development. However, this paper aims to focus only on SED for DHH students that relate to communication, language development, and the life experience of being DHH individuals in the hearing world. Different research shows that DHH students are more likely to experience social difficulties than their hearing peers (Bain et al., 2004; Batten et al., 2013; Israelite et al., 2002; Keating and Mirus, 2003).

### Social–emotional competencies

Research for SED has been extensive in recent years (Ashdown and Bernard, 2012; Bierman et al., 2014; Godoy et al., 2019; Whitcomb and Merrell, 2011). Therefore, many schools include social and emotional learning in curricula, policies and practices (Osher et al., 2016). These programs and practices that related to SED provide an inclusive background that promotes caring relationships, youth voice, agency, and character, and strengthening school-family-community partnerships to support student development (Mahoney et al., 2020).

Social development encompasses a person’s social skills, their standing among peers, and their ability to form interpersonal relationships. These factors contribute to overall social outcomes throughout a person’s life. Essentially, it’s about how an individual learns to interact with others and navigate their social world (Niu et al., 2025). However, this is just one perspective on SED, and this section explores various definitions. Many definitions emphasize socially acceptable behaviors as central to social development (Stefan, 2008). Emotional development, on the other hand, involve the ability to express, regulate, and recognize emotions (Denham, 2006; Stefan, 2008). Therefore, SED is a comprehensive concept, as emotional development is built on social development, and social development is essential for developing emotional skills. Emotional engagement occurs with social interaction (Parkinson et al., 2005), as individuals must be able to manage, express, and understand both their emotions and the emotions of others. These emotional skills are necessary for demonstrating appropriate social behavior, behaving properly in social situations, and in relationship with others (Halberstadt et al., 2001).

The literature discussed various definitions of SED. Two researchers have established a well-developed definition that provides a clear and comprehensive understanding of SED (Denham, 2006; Wigelsworth et al., 2010). The definition of SED covers five important skills: behavioral and emotion regulation, emotion knowledge, social and relationship skills, social problem solving, and emotional expressiveness (see Table 1) (Denham, 2006; Wigelsworth et al., 2010).

**Table 1:** Definitions of core social–emotional development skills (Denham, 2006; Wigelsworth et al., 2010).

SED skills	Definitions
Behavioral and emotion regulation	refers to the ability to recognize, adjust, enhance, retain, and dampen behaviors and emotions.
Emotion knowledge	Involves developing an understanding of emotional expressions and recognizing emotions in different sittings
Social and relationship skills	The ability to build and maintain positive relationships, cooperate with others, and involve in active listening.
Social problem solving	The ability to identify effective strategies for resolving conflicts or issues both personally and with peers.
Emotional expressiveness	Involves displaying more positive emotions than negative ones in interactions with others.

The literature employs a range of terms to describe SED, often reflecting different aspects of emotional, social, and behavioral skills. The table below includes the terms discussed in the literature (see Table 2). This variation in terminology reflects the multidimensional nature of SED and its importance across different fields, such as education, psychology, and health. Also these terms had a direct link to assessments, tools, and curriculum therefore the current literature in SED did not agree on a comprehensive and clear definition that had a framework to cover multiple skills under SED (Humphrey et al., 2011).

**Table 2:** Summary of social–emotional terms with citations.

Terms	References
Social–emotional development	Ashdown and Bernard (2012); Carter et al. (2004); Whitcomb and Merrell (2011)
Social–emotional competence	Bierman et al. (2014)
Social-emotion learning	Collie et al. (2012); Stillman et al. (2018); Ulupınar et al. (2019)
Wellbeing	Coyle et al. (2014)
Social–emotional skills	Ashdown and Bernard (2012)
Behavioral adjustment	Carter et al. (2004)
Socioemotional functioning	Savina and Wan (2017)

### The purpose

**Figure 1:**
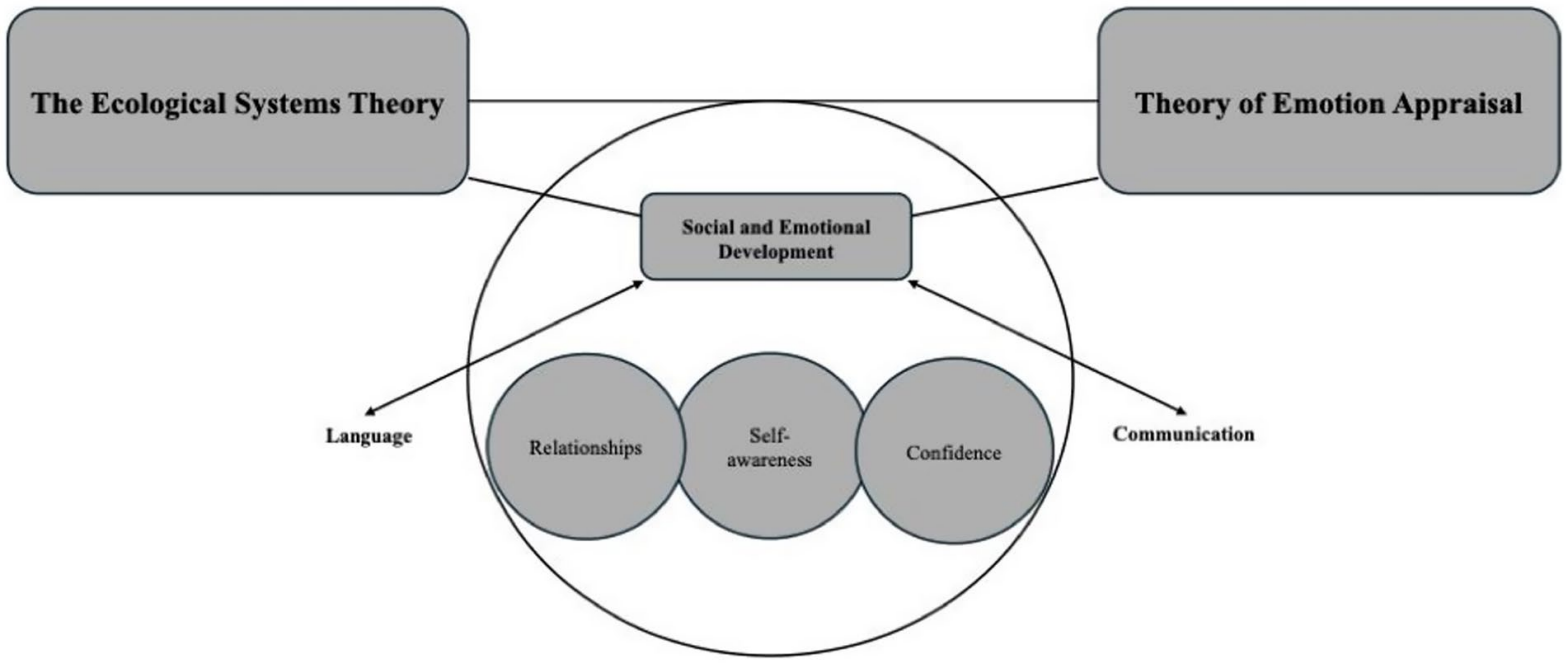
The theoretical framework.

The purpose of this paper is to provide an in-depth analysis of SED in relation to language and communication development among students who are DHH. Grounded in Ecological Systems Theory and the Appraisal Theory of Emotion, this paper explores how hearing loss introduces barriers that significantly impact students who are DHH. Access to language, communication, and related skills is crucial for active social participation, and limited opportunities for social engagement can delay the emotional development of these students. A comprehensive review of research on SED for children and adolescents who are DHH highlights the need for a multidimensional perspective that incorporates educational, medical, linguistic, and psychological approaches. Therefore, this paper examines key factors influencing SED for school-age students who are DHH, specifically focusing on relationship with peers, self-awareness, and confidence please refer to Figure 1.

### Theoretical framework

Understanding human behavior is complex and context-dependent (Burns et al., 2015). Bronfenbrenner (1979) emphasized that individuals cannot be fully understood apart from the environments they live in, such as home, school, peer groups, and community settings. To grasp how individuals grow and function within these settings, it is important to consider the mutual interaction between individuals and their environments (Tudge et al., 2009). The Ecological Systems Theory (EST) is a useful model for understanding how development unfolds across multiple, interconnected environmental levels.

Bronfenbrenner (1979) identified four main environmental levels: the microsystem, mesosystem, exosystem, and macrosystem. The microsystem includes direct settings such as home, school, and peer groups where daily interactions shape development. The mesosystem reflects the relationships between two or more of these settings, such as the connection between school and home. The exosystem refers to environments that indirectly affect individuals, such as parental workplaces or neighborhood dynamics. The macrosystem encompasses broader societal factors like cultural norms, policies, and belief systems that influence all other systems (Onwuegbuzie et al., 2013). This framework has been widely applied in research on children and adolescents with disabilities, including DHH students, particularly to understand their communication and emotional experiences within various environments (Borg et al., 2008; Cawthon et al., 2017; Kohler et al., 2002).

The second guiding theory is the Appraisal Theory of Emotion (ATE), which explores how individuals generate emotional responses to events in their environment. This theory evolved from earlier models like Feedback and Common-Sense theories, offering a deeper look into the connection between emotions and cognitive evaluation (De Ruiter et al., 2020; Gentzler et al., 2010; Scherer et al., 2001; Schreuder et al., 2016). ATE posits that emotional responses are not automatic but result from how a person interprets or appraises a situation. Two people may experience the same event differently depending on their background, personality, and past experiences (Parkinson et al., 2005).

At its core, ATE highlights that emotion is shaped by the individual’s appraisal of social or environmental events. Emotional responses are influenced by factors like interpersonal relationships, cultural values, and group dynamics (Parkinson et al., 2005). Positive social interactions can promote emotional well-being, while negative or exclusionary experiences can lead to distress. This theory has been supported by research showing that social interaction plays a key role in shaping emotions, particularly in group or educational settings (Amodio and Frith, 2006; Van Kleef, 2009). ATE is particularly relevant in understanding how DHH students respond emotionally to communication challenges or social barriers within mainstream environments.

The core principle of ATE is that emotions are not only passive responses but are shaped by the individual’s interpretation and evaluation of events (Troiano et al., 2022). The way individuals assess an event significantly influences their emotional reaction. As such characters and past experiences affect how a person interprets and emotionally engages with any event, leading to unique emotional responses (Parkinson et al., 2005). Social interaction influences emotions in an individual. Therefore, interpersonal encounters and relationships, group relationships, and cultural values impact the emotion of the individual during an event. Research states that normal social interaction leads to appropriate emotion (Amodio and Frith, 2006; Van Kleef, 2009).

## Method

### Protocol and eligibility criteria

**Figure 2:**
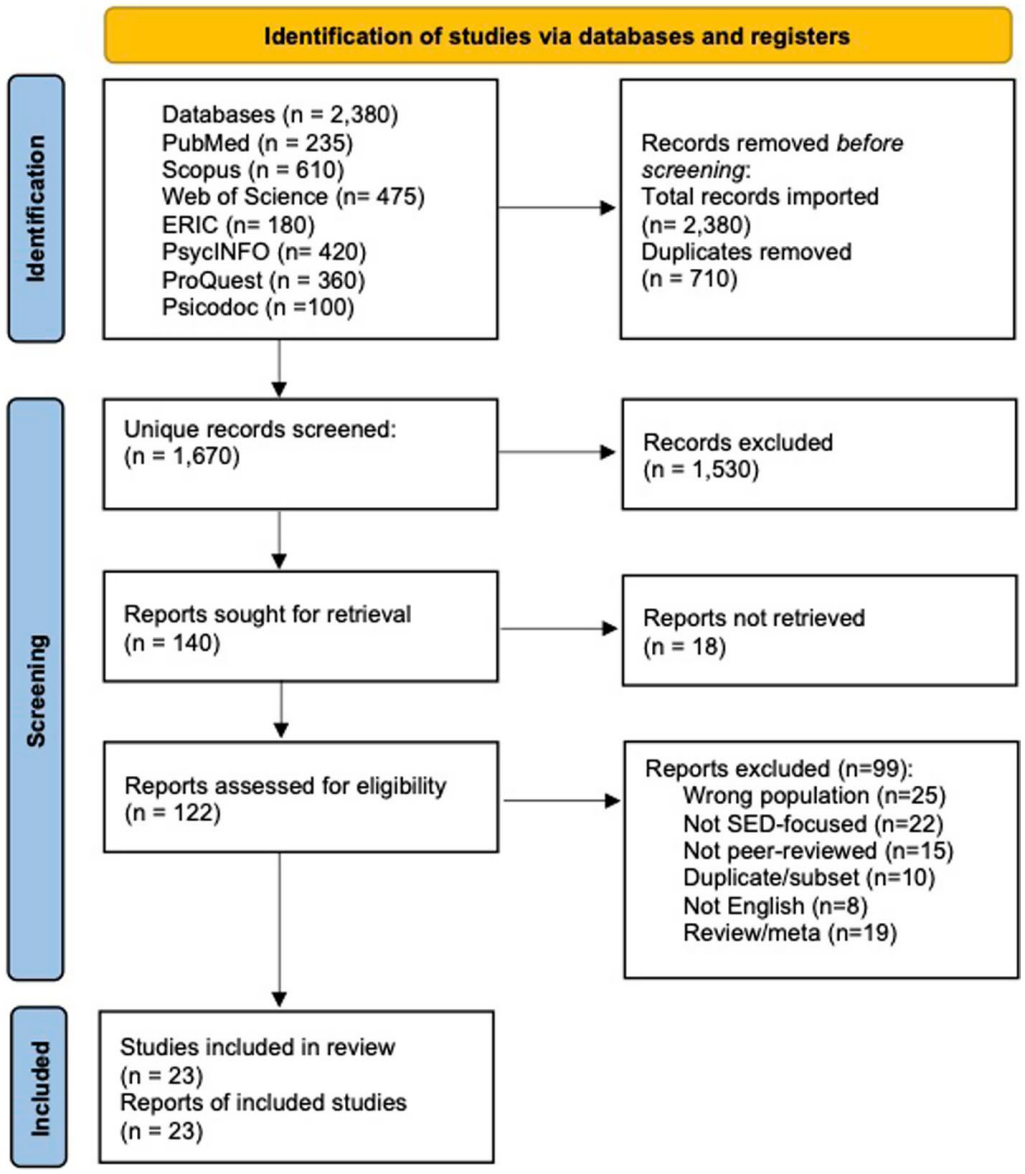
PRISMA guidelines.

This systematic review was conducted following the Preferred Reporting Items for Systematic Reviews and Meta-Analyses (PRISMA) guidelines please refer to Figure 2. To be included in the review, articles had to meet the following criteria: (a) be original empirical studies (qualitative, quantitative, or mixed-methods), including peer-reviewed journal articles and doctoral dissertations; (b) be written in English and published between 2010 and 2025; (c) focus on Deaf or Hard of Hearing (DHH) individuals across any age group (from preschool to adulthood); (d) examine at least one aspect of social–emotional development or learning, such as emotional regulation, self-awareness, peer relationships, self-esteem, identity development, or emotional well-being; (e) address language access, communication methods (e.g., sign language, spoken communication), or barriers affecting social–emotional outcomes; and (f) use validated measurement tools or established qualitative frameworks for assessing social–emotional variables. The following types of studies were excluded: (a) literature reviews, theoretical papers, book chapters, conference abstracts, opinion pieces, or editorials; (b) studies that did not include DHH participants or only addressed broader disability populations without specific findings for DHH individuals; (c) articles not written in English or published outside the 2010–2025 date range; (d) studies focusing exclusively on language acquisition, academic achievement, or medical/audiological interventions without linking to social–emotional outcomes; and (e) studies that did not report methods of data collection clearly, lacked participant demographics, or did not demonstrate validity or reliability in measurement or analysis.

### Search strategy

The search strategy was designed to identify peer-reviewed empirical studies that focused on social–emotional development and learning in DHH individuals. The literature search was conducted on July 14, 2025. To ensure a comprehensive review, seven databases were systematically searched: PubMed, Scopus, Web of Science, ERIC, PsycINFO, ProQuest Psychology, and Psicodoc. The searches were limited to publications from January 2010 to July 2025 and restricted to studies written in English. All types of empirical research were included (qualitative, quantitative, and mixed-methods), while reviews, theoretical articles, and opinion papers were excluded.

A combination of Boolean operators and relevant keywords was used to guide the search across titles, abstracts, and keywords. The core search string used across databases was:

(“Deaf” OR “Hard of Hearing” OR “hearing loss” OR “DHH” OR “d/Deaf”)
AND
(“social emotional development” OR “SED” OR “social–emotional” OR “emotional growth” OR “emotional development” OR “social skills” OR “social competence”)
AND
(“language development” OR “language access” OR “communication methods” OR “sign language” OR “spoken language” OR “communication barriers”)
AND
(“self-awareness” OR “confidence” OR “self-esteem” OR “identity development” OR “personal identity”)
AND
(“peer interaction” OR “peer relationship” OR “social participation” OR “social integration”)

The Boolean logic was adapted slightly depending on the platform’s syntax requirements. A total of 2,380 records were retrieved from all databases: PubMed (n = 235), Scopus (n = 610), Web of Science (n = 475), ERIC (n = 180), PsycINFO (n = 420), ProQuest Psychology (n = 360), and Psicodoc (n = 100). These records were imported into Zotero for reference management and deduplication, which resulted in 710 duplicate records being removed. The remaining 1,670 unique records were then screened for relevance using predefined inclusion and exclusion criteria.

### Study selection

The study selection process followed a systematic approach to identify, screen, and include relevant empirical articles. A total of 2,380 records were retrieved from database searches. After importing all records into the Zotero reference manager and removing 710 duplicates, 1,670 unique articles remained for screening. The author conducted a two-step screening process: an initial screening based on titles, followed by a detailed review of abstracts using pre-established inclusion and exclusion criteria. To assess the consistency of the selection process, the Cohen’s Kappa concordance index (Landis and Koch, 1977) was calculated for the abstract screening stage and indicated substantial agreement (*κ* = 0.78). Discrepancies in classification during the pilot phase were resolved in accordance with the eligibility criteria.

Subsequently, 140 full-text articles were reviewed for eligibility. From the full-text articles assessed, 18 could not be retrieved, and 99 were excluded due to reasons such as lack of focus on social–emotional development, non-DHH populations, insufficient methodological rigor, or being systematic reviews or meta-analyses. An additional two studies were identified through manual searches. In total, 23 empirical studies were included in the final review, encompassing a diverse range of age groups, geographic settings, and methodological designs relevant to the social–emotional development of DHH individuals.

## Results

### Characteristics of the studies

The 23 studies included in this systematic review spanned a variety of research designs, participant demographics, and geographical settings. In terms of methodology, 65.2% (n = 15) of the studies used quantitative designs, such as cross-sectional surveys, longitudinal studies, and experimental interventions. 26.1% (n = 6) employed qualitative methods, including interviews and document analysis. The remaining 8.7% (n = 2) utilized mixed-methods approaches, combining quantitative and qualitative techniques (see Table 3).

**Table 3:** Selected studies.

Reference	Country	Methodology	Sample size	Age group	Communication mode
1. Alsayed (2024)	Saudi Arabia	Qualitative (Interviews)	18 teachers	Professionals (teachers)	Spoken language
2. Antia et al. (2011)	United States	Longitudinal Quantitative	191 students	Grades 2–8 (approx. 7–14 yrs)	Mixed (72% spoken, 8% sign only, rest combined)
3. Brown and Cornes (2015)	Australia	Quantitative (Survey)	89 deaf and hard-of-hearing (DHH)	Adults (various ages)	Mixed (signers, spoken – diverse identities)
4. Brunnberg (2010)	Sweden	Qualitative (Longitudinal)	29 children	Preteens (9–16 years)	Transition from spoken to bilingual (sign + speech)
5. Byatt et al. (2023)	Australia	Qualitative (IPA interviews)	10 adolescents	Adolescents (13–16 yrs)	Mixed (some sign-language users, some spoken)
6. Carter and Mireles (2016)	United States	Quantitative (Survey)	346 deaf individuals	Adult	Mixed communication mode
7. Chen et al. (2025)	United States	Mixed method	20 DHH 20 Hearing students	college students	Sign language
8. Cheng et al. (2025)	China	Quantitative (Survey)	214 deaf or hard-of-hearing (DHH)	secondary schools for the deaf	Mixed communication mode
9. Da Silva et al. (2022)	Portugal	Quantitative	12 preschool DHH children 85 typically hearing (TH) peers	Preschool	Spoken language
10. Hendry et al. (2020)	United Kingdom	Qualitative	16	college students and college graduates	Sign language
11. Hoffman et al. (2015)	United States	Quantitative (Comparative)	74 DHH and 38 hearing	Preschool children (2.5–5 years)	Spoken language (with hearing aids/CIs)
12. Jackson (2011)	United States	Quantitative (Survey)	456 family	Family of children who are DHH	Mixed (spoken, sign—varied)
13. Laugen et al. (2017)	Norway	Quantitative	14 children with UMHL 21 children with MSHL 123 children with TH	4–5 years old	Spoken language
14. Lesar and Smrtnik Vitulić, (2014)	Slovenia	Quantitative (Survey)	80 D/HH students	regular and special primary schools (grades 6–9) and from regular and special secondary schools (grades 1–4)	N/A
15. Martin et al. (2011)	United States	Quantitative (experimental study)	10 children (with CI)	Early childhood (5–6 yrs)	Spoken language (auditory–spoken CI users)
16. Mathews (2024)	Ireland	Quantitative (Survey, Nationwide)	113 students	students (4–17 years)	Mixed (spoken, sign – varied DHH backgrounds)
17. Norman and Jamieson (2015)	United States	Mixed method (Survey and Interviews)	itinerant teachers of the deaf and hard of hearing (ITDHHs)	Itinerant teachers	N/A
18. Rieffe (2012)	Netherlands	Quantitative (Experimental)	26 DHH and 26 hearing	Children (~11 years)	Primarily spoken (spoken communication)
19. Rosas et al. (2023)	Chile	Quantitative	23 deaf 29 blind	primary education students	Mixed (sign and spoken language)
20. Begon (2024)	Germany	Qualitative (Document Analysis)	Six teachers of the d/Deaf and hard-of-hearing	Elementary students	Spoken language
21. Strobel et al. (2023)	Norway (and Sweden)	Quantitative (Cross-sectional)	106 children	Children and adolescents (6–16 yrs)	Mixed (signing Deaf vs. spoken HoH compared)
22. Terlektsi et al. (2020)	United Kingdom	Qualitative (Interviews)	30 adolescents	Adolescents (13–19 years)	Mixed (sign and spoken, mainstream and deaf settings)
23. Warner-Czyz et al. (2015)	United States	Quantitative (Survey)	50 children	Children and adolescents (~8–18 yrs)	Spoken language (CI or HA users)

The studies covered a wide range of participant groups. A total of 17 studies focused on children and adolescents, with 4 of them placing greater emphasis on preschool-aged children. In addition, 6 studies involved adults or professionals, offering broader perspectives on social–emotional development across different stages of life.

The research was conducted across 13 countries. The United States contributed the largest number of studies with 8 studies (34.8%). Other countries included Australia (2 studies), Norway (2 studies), Sweden (2 studies), and the United Kingdom (2 studies). Single studies were conducted in China, Chile, Germany, Ireland, the Netherlands, Portugal, Saudi Arabia, and Slovenia.

The studies also reflected a diversity in communication among participants. Specifically, 34.8% (n = 8) of the studies focused on spoken language users, while 47.8% (n = 11) included bimodal or bilingual communicators. A smaller number, 8.7% (n = 2), focused on sign language users, and the communication mode was not specified for the remaining 8.7% (n = 2).

### Emotional regulation

Research indicates that DHH children often face unique challenges in understanding and managing their emotions. DHH children frequently show delays or differences in emotional awareness and regulation compared to hearing peers, largely due to reduced access to incidental communication about feelings (Rieffe, 2012; Mathews, 2024). For example, one study found DHH pre-adolescents were less able to distinguish between different negative emotions and had limited strategies for regulating negative feelings, leading to more prolonged negative moods (Rieffe, 2012). Likewise, population surveys consistently report higher rates of emotional and behavioral difficulties in DHH youth relative to hearing peers, attributable not to deafness per se but to communication barriers and associated stressors (Mathews, 2024). At the same time, when DHH children are raised in highly accessible linguistic environments (e.g., early exposure to sign language or effective amplification), they can develop emotional understanding on par with hearing children (Da Silva et al., 2022). This suggests that many emotional-regulation gaps are environmental rather than inherent.

Importantly, several factors predict better emotional regulation outcomes in DHH children. Early identification and communication access are critical: children who receive early intervention and rich language input (spoken and/or signed) tend to have greater emotion vocabulary and coping skills, which buffers them against emotional difficulties (Hoffman et al., 2015; Mathews, 2024). Family communication plays a role as well. DHH children with families fluent in their communication mode (spoken or sign) can discuss feelings openly and learn to self-regulate more effectively. In addition, targeted educational efforts can improve skills: teachers increasingly set social–emotional goals (e.g., recognizing others’ feelings, self-control strategies) in students’ educational plans (Alsayed, 2024; Begon, 2024). A recent synthesis of intervention research confirmed the promise of direct instruction in emotional skills (e.g., using social stories or modeling of emotions), though it noted the need for more rigorous studies in DHH populations (Luckner and Movahedazarhouligh, 2019). Innovative approaches are also emerging; for instance, technology-based programs (like emotion-recognition apps and games) have been piloted to help DHH students practice identifying facial expressions and emotional cues, showing positive engagement (Chen et al., 2025). Overall, DHH children’s capacity for emotional regulation improves markedly when they have early, consistent communication access and explicit support in learning about emotions. These findings underscore that differences observed between DHH and hearing peers in emotional regulation can be minimized or even eliminated in environments that prioritize accessible communication and emotional learning.

### Peer interaction and relationships

Developing peer relationships is another area where DHH children often experience difficulties relative to hearing peers. Communication barriers in group settings can lead to frustration, isolation, or superficial interactions for DHH students in inclusive environments (Hendry et al., 2020; Terlektsi et al., 2020). Qualitative interviews with DHH adolescents reveal that many have fewer close friendships and more peer problems compared to hearing youth, especially in environments where they are the only deaf student (Terlektsi et al., 2020). These teens described challenges like feeling left out of fast-paced conversations and struggling to initiate interactions with hearing classmates, which sometimes led to loneliness or being perceived as shy. Likewise, studies of younger children have noted lagging social skills in DHH preschoolers that can hinder play and friendship formation; for example, DHH children aged 3–5 were observed to have lower social competence scores than hearing peers, largely due to delays in spoken language affecting their ability to engage with peers (Hoffman et al., 2015). Such findings align with broader evidence that DHH children in inclusive settings often have trouble communicating, entering group play, and maintaining friendships without support (Antia et al., 2011; Bat-Chava and Deignan, 2001). Notably, hearing peers do not face these same communication hurdles, which puts DHH students at a relative disadvantage in building peer connections.

Despite these challenges, many DHH children do form positive peer relationships, and certain factors predict more successful social outcomes. A longitudinal study of DHH students in general education found that, on average, their social skills and problem behaviors were within normal ranges over time, suggesting many adjust well (Antia et al., 2011). The quality of communication access was identified as a key predictor: students who were more actively involved in classroom communication (e.g., using interpreters or hearing devices effectively, and having teachers and classmates who accommodated their needs) had better social skills growth (Antia et al., 2011). Participation in extracurricular activities also consistently predicted positive peer outcomes for DHH students, as it provided structured opportunities to socialize and be seen by peers beyond the academic context (Antia et al., 2011). Additionally, having DHH peers or a supportive peer group makes a significant difference. DHH adolescents who attended schools with other signing deaf students, or who had a close group of friends aware of their hearing loss, reported higher friendship quality and less peer stress than those who were socially isolated in all-hearing settings (Brunnberg, 2010; Terlektsi et al., 2020). Even for cochlear implant users who communicate by using spoken language, socialization can carry an extra cognitive load. Recent research on “interaction fatigue” found that some DHH adolescents become exhausted from listening and speaking in noisy group conversations, which in turn can limit their willingness to engage socially (Du et al., 2024). This highlights the importance of accessible communication environments (such as reduced background noise, use of sign support, or captioning) to sustain DHH students’ social participation. In total, while DHH children may initially have more difficulty forming and maintaining peer relationships, those difficulties can be mitigated through inclusive practices, peer education, and ensuring the child has both the tools and opportunities to communicate on equal footing with classmates.

### Self-awareness and self-esteem

Self-esteem, which refers to a child’s overall perception of their own worth, is widely recognized as essential for healthy development. Among DHH children and adolescents, research on self-esteem shows mixed but instructive results. Some studies in the early 2000s reported that children with hearing loss tended to have lower self-esteem on average than their hearing counterparts, often linking this to feelings of being “different” or experiences of social exclusion (Bat-Chava and Deignan, 2001). For instance, school-age children with cochlear implants in one study demonstrated more negative self-perceptions related to peer acceptance and academic ability than did hearing students, reflecting the extra challenges they faced (Bat-Chava and Deignan, 2001). However, more recent and larger-scale investigations suggest a more nuanced picture. Warner-Czyz et al. (2015) found that overall self-esteem levels in children and adolescents with hearing loss were broadly comparable to hearing peers, especially when those children had good family and educational support. In that study, DHH participants’ average self-esteem scores did not significantly differ from normative hearing samples, indicating that many DHH youth develop a positive self-concept. Yet, even Warner-Czyz et al. noted important variations: teenagers with hearing loss sometimes showed dips in self-esteem during adolescence, coinciding with greater awareness of social differences and any communication difficulties in high school. In contrast, DHH children who felt well-integrated. For example, those who communicated easily with family and friends or participated in Deaf community activities and often reported high self-worth and pride in themselves (Bat-Chava, 2000; Warner-Czyz et al., 2015). These findings underscore that being DHH does not inherently determine self-esteem; rather, self-esteem outcomes are tightly linked to the child’s environment and experiences. Compared to their hearing peers, DHH youth may be more vulnerable to self-esteem challenges if they encounter stigma, isolation, or repeated frustration, but they can also thrive with the right support.

Predictors of better self-esteem in DHH young people include many of the same protective factors noted in other domains. Effective communication and social inclusion are paramount. DHH children who can converse comfortably with family and peers (whether through spoken language, sign, or both) tend to view themselves more positively, as they feel understood and valued (Antia et al., 2011; Strobel et al., 2023). Conversely, those who experience communication breakdowns or chronic isolation (for example, being left out of conversations on the playground) may internalize negative self-concepts. Another important factor is family and school support. Family and school environments play a critical role in shaping self-esteem. When parents adopt a strengths-based perspective toward their child’s deafness and maintain high expectations, children are more likely to develop a positive self-image. Likewise, school settings that promote awareness, inclusion, and proactive peer education can enhance feelings of acceptance and support among DHH students. In addition, identification with Deaf culture or a community of similar peers can reinforce self-esteem. DHH adolescents who embrace a Deaf identity or who have deaf role models frequently describe feeling more secure and proud of who they are, which translates into higher self-esteem (Bat-Chava, 2000; Byatt et al., 2023). Overall, the research suggests DHH children’s self-esteem is highly malleable and contingent on social experiences: with inclusion and affirmation, DHH youth develop as confident and self-aware as any hearing youth, but without such support they are at risk of lower self-concept.

### Identity development

Identity development in DHH children and adolescents is a complex, dynamic process, often involving the navigation between Deaf and hearing worlds. Unlike hearing peers, who typically develop identity without reference to hearing status, DHH individuals must decide how their deafness figures into their sense of self. Early research by Bat-Chava (2000) highlighted the diversity of deaf identities in the community. In a landmark study, Bat-Chava categorized deaf adults’ identities into four general patterns: culturally Deaf, culturally hearing, bicultural, and marginal. Those with a culturally Deaf identity primarily affiliate with the Deaf community, use sign language, and view deafness as a cultural identity; those with a culturally hearing identity strive to assimilate into the hearing world, often minimizing association with Deaf culture; bicultural individuals integrate both Deaf and hearing aspects in their lives; and marginal individuals feel belonging in neither community. Notably, the study found clear links between identity type and personal outcomes. Participants with strong Deaf or bicultural identities tended to report higher self-esteem and life satisfaction, whereas those with a marginal identity (neither strongly Deaf nor hearing-identified) showed the lowest self-esteem and more psychosocial difficulties (Bat-Chava, 2000). This suggests that embracing a positive identity, whether Deaf or bicultural, is protective for development. Adolescents, in particular, go through an important period of identity formation. During this time, many DHH teens explore what being deaf means to them, often seeking out deaf peers or mentors as they shape their identity (Byatt et al., 2023). Indeed, recent qualitative research using interpretive phenomenological analysis found that social networks and “social capital” play a pivotal role in deaf adolescents’ identity construction (Byatt et al., 2023). Teens in that study who had greater social capital such as access to a Deaf community, supportive friends, or family who sign were more confident in their identity and felt a stronger sense of belonging. In contrast, those who lacked peer support or were in exclusively hearing environments often described uncertainty or conflict about their identity (Byatt et al., 2023). These findings reinforce that identity for DHH youth is not formed in isolation; it develops through cultural exposure, community, and acceptance.

Several factors have been identified as predictors of healthier identity development in DHH individuals. Early cultural exposure is one: children introduced to sign language and Deaf cultural events early in life frequently develop a secure Deaf identity or a comfortable bicultural identity, as they see positive examples of deaf adults and peers (Brunnberg, 2010). By contrast, DHH children raised without contact with other deaf individuals may initially internalize a sense of being “other” in a hearing world, which can lead to marginal identity until they discover the Deaf community later (if at all). School setting and peer group also significantly influence identity. DHH adolescents who attend schools for the Deaf or mainstream programs with a critical mass of DHH peers often report a stronger sense of belonging and pride, as they can share experiences and communicate directly with similar peers (Brunnberg, 2010; Terlektsi et al., 2020). In one longitudinal Swedish study, hard-of-hearing students transitioning from spoken mainstream classrooms to a bilingual program (adding sign language) demonstrated improved feelings of inclusion and a clearer identity, suggesting that access to a signing peer group boosted their sense of self (Brunnberg, 2010). Even in mainstream high schools, having opportunities to meet other DHH youth through extracurricular groups or summer camps has been cited as a turning point for many adolescents in embracing their identity. Family attitudes toward deafness are also a significant predictor of identity development. When families promote a positive view of deafness, such as learning sign language together or highlighting Deaf role models, DHH children are more likely to perceive their deafness as a source of strength rather than a limitation. This perspective can support the formation of a healthy bicultural identity that values both Deaf and hearing experiences. In contrast, when deafness is framed primarily as a deficit by families or schools, children may face greater challenges in developing self-acceptance.

Ultimately, research across these studies emphasizes that a positive identity (Deaf or bicultural) is strongly associated with better social–emotional well-being, including higher self-esteem and resilience (Bat-Chava, 2000; Byatt et al., 2023). Therefore, facilitating identity development by providing DHH children with deaf mentors, peer networks, and cultural knowledge is a crucial component of supporting their overall socio-emotional growth.

Across all four themes, the literature converges on the idea that differences between DHH and hearing peers in socio-emotional domains are largely shaped by access and environment. DHH children are most at risk for emotional-regulation difficulties, social isolation, low self-esteem, or identity confusion when they lack communication access and inclusive support. Conversely, when DHH youth have early and consistent language exposure (spoken and/or signed), supportive peers and family, and a positive orientation toward Deafness as a culture, they demonstrate resilience and outcomes comparable to their hearing peers (Hoffman et al., 2015; Mathews, 2024). In fact, many of the “gaps” can be fully bridged with appropriate intervention. The studies reviewed show that predictors such as early intervention, communication-rich schooling, cultural affiliation, and social support are vital in fostering well-adjusted DHH children. Moving forward, it is *important* for educators and clinicians to give equal attention to social–emotional development alongside language and academic skills. This can be achieved by integrating social–emotional goals into individualized education programs (IEPs) (Alsayed, 2024; Begon, 2024) and implementing initiatives that foster peer connections and mentorship opportunities for DHH students. Such efforts can support not only academic achievement but also emotional well-being and social growth, contributing to the development of confident and well-rounded individuals.

## Discussion

### Relationship with peers

These theoretical insights are echoed by findings, which showed that DHH students often experience difficulties forming friendships in mainstream settings due to communication barriers. However, several studies found that when students had access to signing peers, supportive classroom environments, and extracurricular activities, their social outcomes significantly improved (Antia et al., 2011; Terlektsi et al., 2020). Furthermore, innovative interventions such as the use of interpreters and structured peer groups were associated with improved peer interactions. These findings support the notion that challenges in peer connection are not inevitable but are shaped by environmental and communicative access.

The need to feel connected to others, or a sense of relatedness, is a fundamental human need. Along with relatedness, autonomy and competence are essential components of well-being (Osterman, 2000). The way individuals connect with others significantly influences their emotions and behavior on both intrapersonal and interpersonal levels (Furrer and Skinner, 2003). Research indicates that facilitating communication between individuals or within communities can be more challenging than implementing behavioral or cognitive support plans (Osterman, 2000). Building meaningful relationships and engaging with the world are crucial aspects of psychosocial development, fostering emotional growth and social well-being (Cawthon et al., 2018). Understanding the desire for a sense of relatedness entails being aware of each person’s need to feel firmly a part of and connected to the community (Furrer and Skinner, 2003). The term “community” can refer to a geographical entity, such as a school, neighborhood, or organizational group, or it can imply the nature and traits of interpersonal connections (Osterman, 2000). Relationships within the school community, particularly those with instructors and peers, are part of the need for a sense of relatedness. Social development, emotional stability, and a sense of belonging are all enhanced by these connections (Odescalchi et al., 2024).

DHH Students differ from their hearing peers in terms of their identity, educational options, linguistic barriers, and interpersonal experiences. This can be because they have distinct opportunities for social development (Batten et al., 2013; Cawthon et al., 2018; Duncan et al., 2021; Luckner and Muir, 2001; Terlektsi et al., 2020). These characteristics are connected to various environmental layers that influence the individual. Interpersonal experiences and identity are related to a microsystem, the educational experience can be related to both a mesosystem and exosystem, and the linguistic skills are connected with all environmental circles in Ecological Systems Theory.

The sense of connectedness with peers represents an essential aspect of students’ developmental experiences. Engagement in peer interaction is primarily motivated by the need for psychological well-being (Podiya et al., 2025). Moreover, such interactions facilitate students’ successful adjustment to the school context (Tikkanen et al., 2024). Well-being is an emotional skill (Coyle et al., 2014) which is a part of the umbrella of SED as the paper addresses at the beginning. Well-being relates to the Appraisal Theory of Emotion and the theory considers that human emotion interacts with social events (Scherer et al., 2001). The school adjustment relates to the Ecological Systems Theory. The school adjustment is a skill under the first and second environmental levels, which are microsystem and mesosystem (Bronfenbrenner, 1979). Interpersonal relationships with peers at school represent a fundamental need for belonging and social recognition. This need becomes especially pronounced during adolescence (Ahmadi et al., 2020). Developing healthy social skills is a result of being socially active in the community around the individual (Horst et al., 2007; Mouratidis and Sideridis, 2009; Rodkin et al., 2013; Ryan and Shim, 2006). The peer’s relationship at school is a great example of the students’ community (Osterman, 2000).

DHH Students may have difficulty establishing a friendship with hearing peers, because of the different methods of communication and language development (Antia et al., 2009; Paatsch and Toe, 2020). The methods of communication and language development are strongly related to the first theory of this paper, which is the Ecological Systems Theory, and under the first and second environmental levels being the microsystem and mesosystem. When students who are DHH have difficulty adjusting to the microsystem and mesosystem environmental levels, these students may experience social–emotional difficulties. These social–emotional difficulties are related to the second theory which is the Appraisal Theory of Emotion.

One key factor influencing relationships between DHH students and their hearing peers is language development. Research shows that DHH students often experience vocabulary delays of 1 to 4 years, which can make it more difficult to engage in conversations and build friendships with hearing peers (Bat-Chava, 2000; Davis et al., 2002). These communication challenges may lead to social withdrawal, reduced peer interaction, and feelings of isolation.

While strong communication skills are often linked to more positive peer relationships, some studies present a more complex picture. Research has shown that many DHH students face isolation and social withdrawal due to communication breakdowns with hearing peers. For example, some students reported that verbal attempts to engage in conversation with hearing classmates often ended prematurely due to limited mutual understanding (Keating and Mirus, 2003; Nunes et al., 2001). These findings illustrate that poor verbal communication skills can be a major barrier to forming strong peer connections. Although some research has suggested that DHH students with good spoken skills may maintain fair relationships with hearing peers, others emphasize that when communication gaps persist, students are more likely to feel excluded and disconnected (Keating and Mirus, 2003; Martin and Bat-Chava, 2003; Nunes et al., 2001).

In addition to communication skills, environmental and educational placement factors significantly influence peer relationships for DHH students. As inclusive education has expanded, many DHH students now spend a substantial amount of time in general education classrooms, which form a key part of their social environment (Antia et al., 2009; Gallaudet Research Institute, 2006). However, these settings are not always supportive. Studies indicate that DHH students often struggle to engage with hearing peers due to classroom noise and lack of accessible communication strategies, which can lead to reduced social participation and isolation (Antia et al., 2009; Hilton et al., 2013; Martin et al., 2011). Some research also shows that DHH students feel more comfortable building friendships with other DHH peers, but these opportunities are limited due to the small number of DHH students within mainstream settings (Punch and Hyde, 2011; Wauters and Knoors, 2008). Consequently, many DHH students in inclusive environments experience social withdrawal and find it difficult to form meaningful peer relationships.

Beyond classroom structure and communication modes, the social perception of difference also impacts how DHH students interact with peers. Visible markers such as hearing aids or sign language use may lead to stigmatization, making some students feel “othered” in their school communities (Kouwenberg et al., 2012). Several studies have linked these experiences to reduced life satisfaction and lower quality of peer relationships (Gilman et al., 2004; Rieffe, 2012). Feelings of loneliness, rejection, and emotional isolation are common, and have been identified as forms of psychological distress in DHH students (Coll et al., 2009; Willis and Vernon, 2002). These findings highlight that for many DHH students, the combined effects of communication challenges, unsupportive environments, and social stigma contribute to significant emotional and interpersonal difficulties.

### Self-awareness

The ability to understand one’s own identity and how it places one in the community is known as self-awareness (Bender et al., 2010). Therefore, self-awareness includes race, ethnicity, gender identity, sexual orientation, physical abilities, and cultural background. From the definition of self-awareness, this skill focuses on the identity of the individual. For DHH students, self-awareness is an important SED skill because their self-perception differs from that of hearing students (Hauser et al., 2010).

#### Identity and disability

This aligns with evidence from the reviewed studies, which revealed that students who developed a secure Deaf or bicultural identity often through early exposure to sign language and Deaf role models reported higher self-esteem and fewer psychosocial difficulties (Bat-Chava, 2000; Byatt et al., 2023). Conversely, those in environments lacking linguistic or cultural support more often struggled with marginal identities. The importance of school and family support was also emphasized across multiple studies, with identity development often correlating with social belonging and participation. The study by Carter and Mireles (2016) found that a deaf person’s self-esteem is significantly predicted by the difficulty they experience in verifying their identity in social situations. The more trouble a person has being themselves, the lower their self-esteem tends to be, which supports the hypothesis that difficulty in verifying self-meanings is negatively related to self-esteem. Education was the only other significant predictor across all analyses, with more educated deaf individuals reporting higher self-esteem. Additionally, the study’s bivariate correlations indicated that a stronger identification with being deaf and with the Deaf community is positively associated with higher self-esteem, which aligns with previous research on the benefits of social integration and in-group belonging.

Identity and disability is the first area under self-awareness. Identity is a necessary non-cognitive skill that impacts students’ behaviors and attitudes to be part of the community and school interaction (Flores-Crespo, 2007). The identity formation occurs later on during school age when adolescents have opportunities to identify their own goals, interests, and the way that students interact with their social environment (Good and Adams, 2008). The microsystem environmental level in the Ecological Systems Theory focuses on the immediate interaction between the individual and the environment that is close to the individuals (Bronfenbrenner, 1979). School is a great example of the microsystem.

Research has shown that DHH students are often aware of their disability but may perceive themselves as “less than normal,” not due to the disability itself, but because of negative social interactions within school environments (Kent, 2003, 2006). The development of a positive self-identity is heavily influenced by the immediate environment including home, school, and peers aligning with the Ecological Systems Theory, particularly the microsystem and mesosystem levels (Dalton, 2013). Supportive environments help foster a sense of competence and belonging, while unsupportive settings may reinforce stigma or feelings of inadequacy.

The concept of disability is also shaped by cultural identification. Deaf individuals who are part of the Deaf community often do not consider themselves disabled, whereas hard of hearing students are more likely to self-identify as having a disability (Kent, 2003). Studies show that hard of hearing adolescents who experience supportive school environments tend to have more positive self-perceptions, while those in non-supportive contexts report loneliness, rejection, and emotional distress (Diehl et al., 2018; Israelite et al., 2002; Kent, 2006). These outcomes highlight how school and peer interactions can either support or undermine identity development, consistent with the Appraisal Theory of Emotion, which links emotional responses to social contexts (Parkinson et al., 2005).

#### Culturally identification

Cultural identification plays a significant role in shaping self-awareness among DHH students. Deaf students who are part of the Deaf community often do not view themselves as disabled, as they find acceptance and identity within a shared linguistic and cultural group that uses sign language (Dalton, 2013; Woodcock and Pole, 2008). In contrast, hard of hearing students who lack this community may struggle with self-identity, especially when comparing themselves to hearing peers (Hintermair, 2008). Environments that restrict communication can make even Deaf students feel isolated and helpless, particularly when they are in settings where the majority of people can hear. In these situations, Deaf students may struggle to connect with their peers and participate in group activities, leading to feelings of loneliness and exclusion. The communication barriers can create a sense of powerlessness, as they may feel unable to express their thoughts, needs, or emotions effectively (Silvestri and Hartman, 2022). These findings underscore how cultural belonging and peer context strongly influence the development of self-concept, aligning with the Ecological Systems Theory, which highlights the role of environmental and social systems in shaping identity.

### Confidence

Self-confidence, a key component of social–emotional development (SED), refers to how individuals perceive their abilities, strengths, and value within their social and academic environments (Fox and Sokol, 2011; Stillman et al., 2018). According to both the Ecological Systems Theory and the Appraisal Theory of Emotion, confidence is shaped by interactions with family, peers, school, and broader societal influences. In line with these theories, the systematic review revealed that DHH students’ confidence was closely tied to their communication environment and access to inclusive settings. Studies consistently showed that DHH students in mainstream classrooms with adequate support especially those with consistent access to language demonstrated stronger self-worth and social competence than peers in isolated or unsupported settings.

Several studies highlighted the role of communication competence and peer interaction in shaping confidence. For example, DHH students in inclusive environments often reported higher self-esteem than those in segregated schools, particularly when exposed to spoken language and opportunities for peer engagement (Choi et al., 2020; Lesar and Smrtnik Vitulić, 2014; Martin et al., 2011). However, many DHH students still experience lower confidence than their hearing peers, often due to limited communication skills or misunderstandings with classmates (Silvestre et al., 2007; Lukomski, 2007). DHH students who have better spoken language skills may feel more confident because it allows them to more easily navigate the hearing world. Additionally, other research referenced in the study suggests that developing bilingual skills, encompassing both sign and spoken language, can lead to increased self-esteem and confidence (Lesar and Smrtnik Vitulić, 2014). Research also indicated that hearing students may lack awareness of how to effectively interact with DHH peers, leading to mutual discomfort and social withdrawal (Punch and Hyde, 2005). These challenges are especially prominent among students with moderate to profound hearing loss, who frequently report social isolation and avoidance behaviors in mainstream settings.

Further evidence from qualitative studies supports this trend. Adults reflecting on their school experiences as DHH students reported feelings of isolation and a lack of self-confidence, attributing these issues to insufficient communication support in inclusive classrooms (Bain et al., 2004). Similar findings emerged in research on current students, where those in unsupported environments reported giving up on trying to interact with hearing peers (Thomas, 2018; Wolters et al., 2011). The Appraisal Theory of Emotion suggests that such emotional outcomes stem from how individuals evaluate their experiences in social settings (Amodio and Frith, 2006; Van Kleef, 2009). Taken together, the literature shows that when DHH students face communication barriers and limited inclusion, they are more likely to experience emotional distress, lower confidence, and reduced social engagement (Bain et al., 2004; Thomas, 2018; Wolters et al., 2011).

## Conclusion

This systematic review has provided new insights into the social–emotional development of DHH individuals, focusing on emotional regulation, peer interactions, self-esteem, identity, and confidence. While a considerable body of work has examined language and academic outcomes, fewer studies have specifically explored the socio-emotional dimensions of DHH development across different age groups. The findings from this review emphasized that many of the challenges experienced by DHH children and adolescents are shaped by barriers to communication access and inclusion, rather than by hearing loss itself.

The review also highlighted the importance of the environment in which DHH individuals grow up, including early language exposure, supportive family and school contexts, and opportunities to engage with both hearing and DHH peers. These factors were consistently linked to stronger self-esteem, healthier identity development, and more positive peer relationships. The presence of accessible communication methods and inclusive educational practices emerged as critical facilitators, while the absence of these supports often led to isolation, reduced confidence, and identity struggles.

This review highlights the need for further research that investigates not only barriers but also the effectiveness of targeted interventions and inclusive practices. Future studies should focus on exploring how structured peer programs, mentorship, and cultural belonging shape long-term socio-emotional outcomes for DHH individuals.

## References

[ref1] Ahmadi S. Hassani M. Ahmadi F. (2020). Student- and school-level factors related to school belongingness among high school students. Int. J. Adolesc. Youth 25, 741–752. doi: 10.1080/02673843.2020.1730200

[ref2] Alsayed A. (2024). Social emotional goals for students who are deaf and hard of hearing: special education teacher interviews. Cogent Educ. 11:Article 2422738. doi: 10.1080/2331186X.2024.2422738

[ref3] Amodio D. M. Frith C. D. (2006). Meeting of minds: the medial frontal cortex and social cognition. Nat. Rev. Neurosci. 7, 268–277. doi: 10.1038/nrn1884, PMID: 16552413

[ref4] Antia S. D. Jones P. Luckner J. Kreimeyer K. H. Reed S. (2011). Social outcomes of students who are deaf and hard of hearing in general education classrooms. Except. Child. 77, 489–504. doi: 10.1177/001440291107700407

[ref5] Antia S. D. Jones P. B. Reed S. Kreimeyer K. H. (2009). Academic status and progress of deaf and hard-of-hearing students in general education classrooms. J. Deaf. Stud. Deaf. Educ. 14, 293–311. doi: 10.1093/deafed/enp009, PMID: 19502625

[ref6] Antia S. D. Kreimeyer K. H. (2015). Social competence of deaf and hard-of-hearing children. New York, NY: Oxford University Press.

[ref7] Ashdown D. M. Bernard M. E. (2012). Can explicit instruction in social and emotional learning skills benefit the social-emotional development, well-being, and academic achievement of young children? Early Child. Educ. J. 39, 397–405. doi: 10.1007/s10643-011-0481-x

[ref8] Atkins J. Vega-Uriostegui T. Norwood D. Adamuti-Trache M. (2023). Social and emotional learning and ninth-grade students’ academic achievement. J. Intelligence 11:185. doi: 10.3390/jintelligence11090185, PMID: 37754913 PMC10532598

[ref9] Bain L. Scott S. Steinberg A. G. (2004). Socialization experiences and coping strategies of adults raised using spoken language. J. Deaf. Stud. Deaf. Educ. 9, 120–128. doi: 10.1093/deafed/enh001, PMID: 15304407

[ref10] Bat-Chava Y. (2000). Diversity of deaf identities. Am. Ann. Deaf 145, 420–428. doi: 10.1353/aad.2012.0176, PMID: 11191821

[ref11] Bat-Chava Y. Deignan E. (2001). Peer relationships of children with cochlear implants. J. Deaf. Stud. Deaf. Educ. 6, 186–199. doi: 10.1093/deafed/6.3.186, PMID: 15451849

[ref12] Batten G. Oakes R. M. Alexander T. (2013). Factors associated with social interactions between deaf children and their hearing peers: a systematic literature review. J. Deaf. Stud. Deaf. Educ. 19, 285–302. doi: 10.1093/deafed/ent052 24222193

[ref004] Begon S. (2024). Language education of immigrant d/Deaf and hard-of-hearing multilingual learners – an interview study. J. Multiling. Multicult. Dev. 46, 132–147. doi: 10.1080/01434632.2024.2380818

[ref13] Bender K. Negi N. Fowler D. N. (2010). Exploring the relationship between self-awareness and student commitment and understanding of culturally responsive social work practice. J. Ethn. Cult. Divers. Soc. Work 19, 34–53. doi: 10.1080/15313200903531990, PMID: 23255873 PMC3523726

[ref14] Bierman K. L. Nix R. L. Heinrichs B. S. Domitrovich C. E. Gest S. D. Welsh J. A. . (2014). Effects of head start REDI on children's outcomes 1 year later in different kindergarten contexts. Child Dev. 85, 140–159. doi: 10.1111/cdev.12117, PMID: 23647355 PMC3740043

[ref15] Borg E. Bergkvist C. Olsson I. Wikström C. Borg B. (2008). Communication as an ecological system. Int. J. Audiol. 47, S131–S138. doi: 10.1080/14992020802307362 19012122

[ref16] Boyd J. Barnett W. S. Bodrova E. Leong D. J. Gomby D. (2005). Promoting children’s social and emotional development through preschool education. New Brunswick, NJ: National Institute for Early Education Research.

[ref17] Bronfenbrenner U. (1979). The ecology of human development: Experiments in nature and design. Cambridge, MA: Harvard University Press.

[ref18] Brown P. Cornes A. (2015). Mental health of deaf and hard-of-hearing adolescents: what the students say. J. Deaf. Stud. Deaf. Educ. 201, 75–81. doi: 10.1093/deafed/enu031 25237152

[ref19] Brunnberg E. (2010). Hard-of-hearing children’s sense of identity and belonging. Scand. J. Disabil. Res. 12, 179–197. doi: 10.1080/15017410903309102

[ref20] Burns M. K. Warmbold-Brann K. Zaslofsky A. F. (2015). Ecological systems theory in school psychology review. Sch. Psychol. Rev. 44, 249–261. doi: 10.17105/spr-15-0092.1

[ref21] Byatt T. J. Duncan J. Dally K. (2023). Social capital and identity of d/Deaf adolescents: an interpretive phenomenological analysis. Disabil. Soc. 39, 1961–1983. doi: 10.1080/09687599.2023.2168517

[ref22] Calderon R. Greenberg M. (2011). “Social and emotional development of deaf children: family, school, and program effects” in Oxford handbook of deaf studies, language, and education, vol. 1. eds. Marschark M. Spencer P. E.. 2nd ed (Oxford: Oxford University Press), 188–199.

[ref23] Carter A. S. Briggs-Gowan M. J. Davis N. (2004). Assessment of young children’s social-emotional development and psychopathology: recent advances and recommendations for practice. J. Child Psychol. Psychiatry 45, 109–134. doi: 10.1046/j.0021-9630.2003.00316.x, PMID: 14959805

[ref24] Carter M. J. Mireles D. C. (2016). Exploring the relationship between deaf identity verification processes and self-esteem. Identity 16, 102–114. doi: 10.1080/15283488.2016.1159963

[ref25] Cawthon S. W. Fink B. Schoffstall S. Wendel E. (2018). In the rearview mirror: social skill development in deaf youth, 1990–2015. Am. Ann. Deaf 162, 479–485. doi: 10.1353/aad.2018.0005, PMID: 29479000

[ref26] Cawthon S. W. Fink B. W. K. Tarantolo-Leppo R. H. Wendel E. M. Schoffstall S. J. (2017). Ecological systems and vocational rehabilitation service provision for individuals who are deaf or hard of hearing. J. Appl. Rehabil. Counsel. 48, 32–41. doi: 10.1891/0047-2220.48.2.3

[ref27] Chen S. Situ J. Cheng H. Su S. Kirst D. Ming L. . (2025). Inclusive emotion technologies: addressing the needs of d/Deaf and hard of hearing learners in video-based learning. Proc. ACM Hum.-Comput. Interact. 9:CSCW115. doi: 10.1145/3711013

[ref28] Cheng S. Chen X. Deng L. (2025). Self-determination and student engagement among deaf and hard of hearing students. Int. J. Disabil. Dev. Educ. 1–13, 1–13. doi: 10.1080/1034912X.2025.2528206, PMID: 40922668

[ref29] Choi J. E. Hong S. H. Moon I. J. (2020). Academic performance, communication, and psychosocial development of Prelingual deaf children with Cochlear implants in mainstream schools. J. Audiol. Otol. 24, 61–70. doi: 10.7874/jao.2019.00346, PMID: 31995976 PMC7141989

[ref30] Coll K. M. Cutler M. M. Thobro P. Haas R. Powell S. (2009). An exploratory study of psychosocial risk behaviors of adolescents who are deaf or hard of hearing: comparisons and recommendations. Am. Ann. Deaf 154, 30–35. doi: 10.1353/aad.0.0074, PMID: 19569302

[ref31] Collie R. J. Shapka J. D. Perry N. E. (2012). School climate and social–emotional learning: predicting teacher stress, job satisfaction, and teaching efficacy. J. Educ. Psychol. 104, 1189–1204. doi: 10.1037/a0029356

[ref32] Coyle D. Thieme A. Linehan C. Balaam M. Wallace J. Lindley S. (2014). Emotional wellbeing. Int. J. Hum.-Comput. Stud. 72, 627–628. doi: 10.1016/j.ijhcs.2014.05.008

[ref33] Da Silva B. M. S. Rieffe C. Frijns J. H. M. Sousa H. Monteiro L. Veiga G. (2022). Being deaf in mainstream schools: the effect of a hearing loss in children’s playground behaviors. Children 9:1091. doi: 10.3390/children9071091, PMID: 35884074 PMC9318400

[ref34] Dalton C. (2013). Lessons for inclusion: classroom experiences of students with mild and moderate hearing loss. Can. J. Educ. / Rev. Can. Educ. 36, 125–152. Available at: https://journals.sfu.ca/cje/index.php/cje-rce/article/view/1163

[ref35] Davis A. Reeve K. Hind S. Bamford J. (2002). “Children with mild and unilateral hearing impairment” in A sound foundation through early amplification: Proceedings of the second international conference, Chap. 14. eds. Seewald R. Gravel J. (Stafa Switzerland: Phonak, AG), 179–186.

[ref36] De Ruiter J. Poorthuis A. Aldrup K. Koomen H. (2020). Teachers' emotional experiences in response to daily events with individual students varying in perceived past disruptive behavior. J. Sch. Psychol. 82, 85–102. doi: 10.1016/j.jsp.2020.08.005 32988465

[ref37] Denham S. A. (2006). Social-emotional competence as support for school readiness: what is it and how do we assess it? Early Educ. Dev. 17, 57–89. doi: 10.1207/s15566935eed1701_4

[ref38] Diehl K. Jansen C. Ishchanova K. Hilger-Kolb J. (2018). Loneliness at universities: determinants of emotional and social loneliness among students. Int. J. Environ. Res. Public Health 15:1865. doi: 10.3390/ijerph15091865, PMID: 30158447 PMC6163695

[ref39] Dowling M. (2014). Young children’s personal, social and emotional development. 4th Edn. New York (NY): Sage Publishing.

[ref003] Du X. Huang T. Wang X. Wu S. Chen X. Jiang J. . (2024). Difficulties in implicit emotion regulation of the deaf college students: An ERP study. Heliyon. 10:e34451. doi: 10.1016/j.heliyon.2024.e34451 39816331 PMC11734079

[ref40] Duncan J. Colyvas K. Punch R. (2021). Social capital, loneliness, and peer relationships of adolescents who are deaf or hard of hearing. J. Deaf. Stud. Deaf. Educ. 26, 223–229. doi: 10.1093/deafed/enaa037, PMID: 33333558

[ref41] Flores-Crespo P. (2007). Ethnicity, identity and educational achievement in Mexico. Int. J. Educ. Dev. 27, 331–339. doi: 10.1016/j.ijedudev.2006.10.011

[ref42] Fox M. Sokol L. (2011). Think confident, be confident for teens: A cognitive therapy guide to overcoming self-doubt and creating unshakable self-esteem. Oakland, California: New Harbinger Publications.

[ref43] Furrer C. Skinner E. (2003). Sense of relatedness as a factor in children's academic engagement and performance. J. Educ. Psychol. 95, 148–162. doi: 10.1037/0022-0663.95.1.148

[ref44] Gallaudet Research Institute (2006). Regional and national summary report of data from the 2006–2007 annual survey of deaf and hard of hearing children and youth. Washington, DC: GRI Gallaudet University.

[ref45] Gentzler A. Kerns K. Keener E. (2010). Emotional reactions and regulatory responses to negative and positive events: associations with attachment and gender. Motiv. Emot. 34, 78–92. doi: 10.1007/S11031-009-9149-X

[ref46] Gilman R. Easterbrooks S. R. Frey M. (2004). A preliminary study of multidimensional life satisfaction among deaf/hard of hearing youth across environmental settings. Soc. Indic. Res. 66, 143–164. doi: 10.1023/B:SOCI.0000007495.40790.85

[ref47] Godoy L. Chavez A. E. Mack R. A. Carter A. S. (2019). “Rating scales for social-emotional behavior and development” in Clinical guide to psychiatric assessment of infants and young children. eds. Godoy L. Chavez A. E. Mack R. A. Carter A. S. (Cham: Springer), 217–251.

[ref48] Good M. Adams G. R. (2008). Linking academic social environments, ego-identity formation, ego virtues, and academic success. Adolescence 43, 221–236. Available at: https://link.gale.com/apps/doc/A181522486/AONE?u=anon~9bbc0b1d&sid=googleScholar&xid=7332ac31 18689098

[ref49] Ha C. (2023). Students’ self-regulated learning strategies and science achievement: exploring the moderating effect of learners’ emotional skills. Camb. J. Educ. 53, 451–472. doi: 10.1080/0305764X.2023.2175787

[ref50] Halberstadt A. G. Denham S. A. Dunsmore J. C. (2001). Affective social competence. Soc. Dev. 10, 79–119. doi: 10.1111/1467-9507.00150, PMID: 40923560

[ref51] Hauser P. C. O’Hearn A. McKee M. Steider A. Thew D. (2010). Deaf epistemology: Deafhood and deafness. Am. Ann. Deaf 154, 486–492. doi: 10.1353/aad.0.0120, PMID: 20415284

[ref53] Hendry G. Hendry A. Ige H. McGrath N. (2020). “I was isolated and this was difficult”: investigating the communication barriers to inclusive further/higher education for deaf Scottish students. Deafness Educ. Int. 23, 295–312. doi: 10.1080/14643154.2020.1818044

[ref54] Hilton K. Jones F. Harmon S. Cropper J. (2013). Adolescents' experiences of receiving and living with sequential cochlear implants: an interpretative phenomenological analysis. J. Deaf. Stud. Deaf. Educ. 18, 513–531. doi: 10.1093/deafed/ent025, PMID: 23744061

[ref55] Hintermair M. (2008). Self-esteem and satisfaction with life of deaf and hard-of-hearing people—a resource-oriented approach to identity work. J. Deaf. Stud. Deaf. Educ. 13, 278–300. doi: 10.1093/deafed/enm054, PMID: 17971343

[ref56] Hintermair M. Sarimski K. Lang M. (2017). Preliminary evidence assessing social-emotional competences in deaf and hard of hearing infants and toddlers using a new parent questionnaire. J. Deaf. Stud. Deaf. Educ. 22, 143–154. doi: 10.1093/deafed/enw070, PMID: 27881483

[ref57] Hoffman M. F. Quittner A. L. Cejas I. (2015). Comparisons of social competence in young children with and without hearing loss: a dynamic systems framework. J. Deaf. Stud. Deaf. Educ. 20, 115–124. doi: 10.1093/deafed/enu040, PMID: 25583707 PMC4447823

[ref58] Horst J. S. Finney S. J. Barron K. E. (2007). Moving beyond academic achievement goal measures: a study of social achievement goals. Contemp. Educ. Psychol. 32, 667–698. doi: 10.1016/j.cedpsych.2006.10.011

[ref59] Humphrey N. Kalambouka A. Wigelsworth N. Lendrum A. Deighton J. Wolpert M. (2011). Measures of social and emotional skills for children and young people: a systematic review. Educ. Psychol. Meas. 71, 617–637. doi: 10.1177/0013164410382896

[ref60] Israelite N. Ower J. Goldstein G. (2002). Hard-of-hearing adolescents and identity construction: influences of school experiences, peers, and teachers. J. Deaf. Stud. Deaf. Educ. 7, 134–148. doi: 10.1093/deafed/7.2.134, PMID: 15451881

[ref61] Jackson C. W. (2011). Family supports and resources for parents of children who are deaf or hard of hearing. Am. Ann. Deaf 156, 343–362. doi: 10.1353/aad.2011.0038, PMID: 22256537

[ref62] Keating E. Mirus G. (2003). Examining interactions across language modalities: deaf children and hearing peers at school. Anthropol. Educ. Q. 34, 115–135. doi: 10.1525/aeq.2003.34.2.115

[ref63] Kent B. (2003). Identity issues for hard-of-hearing adolescents aged 11, 13 and 15 in mainstream settings. J. Deaf. Stud. Deaf. Educ. 8, 315–324. doi: 10.1093/deafed/eng017 15448055

[ref64] Kent B. (2006). They only see it when the sun shines in my ears: exploring perceptions of adolescent hearing aid users. J. Deaf. Stud. Deaf. Educ. 11, 461–476. doi: 10.1093/deafed/enj044, PMID: 16699063

[ref65] Kohler E. Keysers C. Umiltà M. A. Fogassi L. Gallese V. Rizzolatti G. (2002). Hearing sounds, understanding actions: action representation in mirror neurons. Science 297, 846–848. doi: 10.1126/science.1070311, PMID: 12161656

[ref66] Kouwenberg M. Rieffe C. Theunissen S. C. de Rooij M. (2012). Peer victimization experienced by children and adolescents who are deaf or hard of hearing. PLoS One 7:e52174. doi: 10.1371/journal.pone.0052174 23284923 PMC3526587

[ref67] Landis J. R. Koch G. G. (1977). The measurement of observer agreement for categorical data. Biometrics 33, 159–174. doi: 10.2307/2529310, PMID: 843571

[ref68] Laugen N. J. Jacobsen K. H. Rieffe C. Wichstrøm L. (2017). Social skills in preschool children with unilateral and mild bilateral hearing loss. Deafness Educ. Int. 19, 54–62. doi: 10.1080/14643154.2017.1344366

[ref69] Lesar I. Smrtnik Vitulić H. (2014). Self-esteem of deaf and hard of hearing students in regular and special schools. Eur. J. Spec. Needs Educ. 29, 59–73. doi: 10.1080/08856257.2013.849842

[ref70] Luckner J. L. Movahedazarhouligh S. (2019). Social-emotional interventions with children and youth who are deaf or hard of hearing: a research synthesis. J. Deaf. Stud. Deaf. Educ. 24, 1–10. doi: 10.1093/deafed/eny030, PMID: 30418589

[ref71] Luckner J. L. Muir S. (2001). Successful students who are deaf in general education settings. Am. Ann. Deaf 146, 435–446. doi: 10.1353/aad.2012.0202, PMID: 11865574

[ref72] Lukomski J. (2007). Deaf college students' perceptions of their social-emotional adjustment. J. Deaf. Stud. Deaf. Educ. 12, 486–494. doi: 10.1093/deafed/enm008, PMID: 17437957

[ref73] Mahoney J. Weissberg R. Greenberg M. Dusenbury L. Jagers R. Niemi K. . (2020). Systemic social and emotional learning: promoting educational success for all preschool to high school students. Am. Psychol. doi: 10.1037/amp0000701 33030926

[ref74] Maller S. Braden J. (2011). “Intellectual assessment of deaf people: a critical review of core concepts and issues” in The Oxford handbook of deaf studies, language, and education, volume 1. eds. Marschark M. Spencer P.. 2nd ed (New York, NY: Oxford University Press), 473–485.

[ref75] Martin D. Bat-Chava Y. (2003). Negotiating deaf–hearing friendships: coping strategies of deaf boys and girls in mainstream schools. Child Care Health Dev. 29, 511–521. doi: 10.1046/j.1365-2214.2003.00371.x, PMID: 14616909

[ref76] Martin D. Bat-Chava Y. Lalwani A. Waltzman S. B. (2011). Peer relationships of deaf children with cochlear implants: predictors of peer entry and peer interaction success. J. Deaf. Stud. Deaf. Educ. 16, 108–120. doi: 10.1093/deafed/enq037, PMID: 20805230

[ref77] Mathews E. S. (2024). Rates of socio-emotional difficulties among deaf and hard of hearing children in Ireland. Deafness Educ. Int. 27, 284–300. doi: 10.1080/14643154.2024.2420429

[ref78] Mekonnen M. Hannu S. Elina L. Matti K. (2015). Socio-emotional problems experienced by deaf and hard of hearing students in Ethiopia. Deafness Educ. Int. 17, 155–162. doi: 10.1179/1557069X15Y.0000000002

[ref79] Mouratidis A. A. Sideridis G. D. (2009). On social achievement goals: their relations with peer acceptance, classroom belongingness, and perceptions of loneliness. J. Exp. Educ. 77, 285–308. doi: 10.3200/JEXE.77.3.285-308

[ref80] Niu S. J. Niemi H. Yang J. Wang J. Wang H. Li J. . (2025). From play to progress: student learning of social skills with a solution-focused approach. Educ. Sci. 15:218. doi: 10.3390/educsci15020218

[ref81] Norman N. Jamieson J. R. (2015). Social and emotional learning and the work of itinerant teachers of the deaf and hard of hearing. Am. Ann. Deaf 160, 273–288. doi: 10.1353/aad.2015.0024, PMID: 26320750

[ref002] Nunes T. Pretzlik U. Olsson J. (2001). Deaf children’s social relationships in mainstream schools. Deafness Educ Int. 3, 123–136. doi: 10.1179/146431501790560972

[ref82] Odescalchi C. Paleczek L. Gasteiger-Klicpera B. (2024). Primary school teachers´ social-emotional competencies and strategies in fostering the social participation of students with SEBD. Eur. J. Spec. Needs Educ. 40, 393–407. doi: 10.1080/08856257.2024.2370149

[ref83] Onwuegbuzie A. J. Collins K. M. T. Frels R. K. (2013). Foreword: using Bronfenbrenner's ecological systems theory to frame quantitative, qualitative, and mixed research. Int. J. Mult. Res. Approaches 7, 2–8. doi: 10.5172/mra.2013.7.1.2

[ref84] Osher D. Kidron Y. Brackett M. Dymnicki A. Jones S. Weissberg R. (2016). Advancing the science and practice of social and emotional learning. Rev. Res. Educ. 40, 644–681. doi: 10.3102/0091732x16673595

[ref85] Osterman K. F. (2000). Students' need for belonging in the school community. Rev. Educ. Res. 70, 323–367. doi: 10.3102/00346543070003323

[ref86] Paatsch L. Toe D. (2020). The impact of pragmatic delays for deaf and hard of hearing students in mainstream classrooms. Pediatrics 146, S292–S297. doi: 10.1542/peds.2020-0242I, PMID: 33139443

[ref87] Parkinson B. Fischer A. Manstead A. S. R. (2005). Emotion in social relations: cultural, group, and interpersonal processes. New York: Psychology Press.

[ref88] Podiya J. K. Navaneetham J. Bhola P. (2025). Influences of school climate on emotional health and academic achievement of school-going adolescents in India: a systematic review. BMC Public Health 25:54. doi: 10.1186/s12889-024-21268-0, PMID: 39762815 PMC11706050

[ref89] Punch R. Hyde M. (2005). The social participation and career decision-making of hard-of-hearing adolescents in regular classes. Deafness Educ. Int. 7, 122–138. doi: 10.1179/146431505790560365

[ref90] Punch R. Hyde M. (2011). Social participation of children and adolescents with cochlear implants: a qualitative analysis of parent, teacher, and child interviews. J. Deaf. Stud. Deaf. Educ. 16, 474–493. doi: 10.1093/deafed/enr001, PMID: 21372111

[ref92] Rieffe C. (2012). Awareness and regulation of emotions in deaf children. Br. J. Dev. Psychol. 30, 477–492. doi: 10.1111/j.2044-835X.2011.02057. 23039328

[ref93] Rodkin P. C. Ryan A. M. Jamison R. Wilson T. (2013). Social goals, social behavior, and social status in middle childhood. Dev. Psychol. 49, 1139–1150. doi: 10.1037/a0029389, PMID: 22822934

[ref94] Rosas R. Espinoza V. Martinez C. Santa Cruz C. (2023). The paradoxes of inclusion: cognitive and socio-emotional developmental trajectories of deaf and blind primary education students in mainstream and special schools. Front. Educ. doi: 10.3389/feduc.2023.1227178

[ref95] Ryan A. M. Shim S. S. (2006). Social achievement goals: the nature and consequences of different orientations toward social competence. Personal. Soc. Psychol. Bull. 32, 1246–1263. doi: 10.1177/0146167206289345, PMID: 16902243

[ref96] Savina E. Wan K. P. (2017). Cultural pathways to socio-emotional development and learning. J. Relat. Res. 8:e19. doi: 10.1017/jrr.2017.19

[ref97] Scherer K. Schorr A. Johnstone T. (Eds.) (2001). Appraisal processes in emotion: Theory, methods, research. Oxford: Oxford University Press.

[ref98] Schreuder E. Van Erp J. Toet A. Kallen V. (2016). Emotional responses to multisensory environmental stimuli. SAGE Open 6. doi: 10.1177/2158244016630591

[ref99] Silvestre N. Ramspott A. Pareto I. D. (2007). Conversational skills in a semistructured interview and self-concept in deaf students. J. Deaf. Stud. Deaf. Educ. 12, 38–54. doi: 10.1093/deafed/enl011, PMID: 16916891

[ref100] Silvestri J. Hartman M. (2022). Inclusion and deaf and hard of hearing students: finding asylum in the LRE. Educ. Sci. 12:773. doi: 10.3390/educsci12110773

[ref101] Stefan C. A. (2008). Short-term efficacy of a primary prevention program for the development of social-emotional competencies in preschool children. Cognit. Brain Behav. Interdiscip. Inf. Sci. 12, 285–307.

[ref102] Stillman P. Stillman S. B. Martinez L. Freedman J. Jensen A. L. Leet C. (2018). Strengthening social emotional learning with student, teacher, and schoolwide assessments. J. Appl. Dev. Psychol. 55, 71–92. doi: 10.1016/j.appdev.2017.07.010

[ref103] Strobel S. Aanondsen C. M. Jozefiak T. Lydersen S. Rimehaug T. (2023). Deaf and hard-of-hearing children and adolescents’ mental health, quality of life and communication. BMC Psychiatry 23:297. doi: 10.1186/s12888-023-04787-9 37118705 PMC10148557

[ref104] Terlektsi E. Kreppner J. Mahon M. Worsfold S. Kennedy C. R. (2020). Peer relationship experiences of deaf and hard-of-hearing adolescents. J. Deaf. Stud. Deaf. Educ. 25, 153–166. doi: 10.1093/deafed/enz048, PMID: 32048717 PMC7167539

[ref105] Thomas C. L. (2018). *Accommodating deaf and hard of hearing students in the mainstream classroom* (order no. 11016065). Available from ProQuest Dissertations & Theses Global. (2158003612). Available online at: https://unco.idm.oclc.org/login?url=https://search.proquest.com/docview/2158003612?accountid=12832

[ref106] Thompson R. A. Goodman M. (2009). “Development of self, relationships, and socioemotional competence” in Handbook of child development and early education: research to practice. eds. Barbarin O. A. Wasik B. H. (New York: Guilford Press), 147–171.

[ref107] Tikkanen L. Anttila H. Ulmanen S. Pyhältö K. (2024). Peer relationships and study wellbeing: upper secondary students’ experiences. Soc. Psychol. Educ. 27, 3097–3117. doi: 10.1007/s11218-024-09942-y

[ref108] Troiano E. Oberländer L. Klinger R. (2022). Dimensional modeling of emotions in text with appraisal theories: Corpus creation, annotation reliability, and prediction. Comput. Linguist. 49, 1–72. doi: 10.1162/coli_a_00461

[ref109] Tudge J. R. H. Mokrova I. Hatfield B. E. Karnik R. B. (2009). Uses and misuses of Bronfenbrenner's bioecological theory of human development. J. Fam. Theory Rev. 1, 198–210. doi: 10.1111/j.1756-2589.2009.00026.x

[ref110] Ulupınar S. Şenyuva E. Küçük Yüceyurt N. (2019). Does participation of nursing students in social activities affect their social emotional learning skills? Nurse Educ. Today 76, 78–84. doi: 10.1016/j.nedt.2019.01.031, PMID: 30772675

[ref111] Umberson D. Montez J. K. (2010). Social relationships and health: a flashpoint for health policy. J. Health Soc. Behav. 51, S54–S66. doi: 10.1177/0022146510383501, PMID: 20943583 PMC3150158

[ref112] Van Kleef G. (2009). How emotions regulate social life: the emotions as social information (EASI) model. Curr. Dir. Psychol. Sci., 18, 184–188. Available online at: http://www.jstor.org.unco.idm.oclc.org/stable/20696025

[ref113] Warner-Czyz A. D. Loy B. Evans C. Wetsel A. Tobey E. A. (2015). Self-esteem in children and adolescents with hearing loss. Trends Hear. 19, 1–12. doi: 10.1177/2331216515572615 PMC4355008 25755025

[ref001] Wauters L. N. Knoors H. (2008). Social integration of deaf children in inclusive settings. J. Deaf Stud. Deaf Educ. 13, 21–36. doi: 10.1093/deafed/enm028, PMID: 17573356

[ref114] Whitcomb S. A. Merrell K. W. (2011). Understanding implementation and effectiveness of strong start K-2 on social-emotional behavior. Early Child. Educ. J. 40, 63–71. doi: 10.1007/s10643-011-0490-9

[ref115] Wigelsworth M. Humphrey N. Kalambouka A. Lendrum A. (2010). A review of key issues in the measurement of children’s social and emotional skills. Educ. Psychol. Pract. 26, 173–186. doi: 10.1080/02667361003768526

[ref116] Willis R. G. Vernon M. (2002). Residential psychiatric treatment of emotionally disturbed deaf youth. Am. Ann. Deaf 147, 31–37. doi: 10.1353/aad.2012.0124, PMID: 12061189

[ref117] Wolters N. Knoors H. E. T. Cillessen A. H. N. Verhoeven L. (2011). Predicting acceptance and popularity in early adolescence as a function of hearing status, gender, and educational setting. Res. Dev. Disabil. 32, 2553–2565. doi: 10.1016/j.ridd.2011.07.003, PMID: 21840686

[ref118] Woodcock K. Pole J. D. (2008). Educational attainment, labour force status and injury: a comparison of Canadians with and without deafness and hearing loss. Int. J. Rehabil. Res. 31, 297–304. doi: 10.1097/MRR.0b013e3282fb7d4d, PMID: 19008678

[ref119] Zero to Three. (2016). Early connections last a lifetime: early development & well-being. Available online at: https://www.zerotothree.org/early-development (Accessed May 20, 2019)

